# A new digital denture procedure: a first practitioners appraisal

**DOI:** 10.1186/s12903-017-0440-z

**Published:** 2017-12-20

**Authors:** Guillaume Bonnet, Cindy Batisse, Marion Bessadet, Emmanuel Nicolas, Jean-Luc Veyrune

**Affiliations:** 1 Université Clermont Auvergne, CROC, F-63000 Clermont-Ferrand, France; 2 CHU Clermont-Ferrand, Service d’Odontologie, F-63003 Clermont-Ferrand, France

**Keywords:** Digital denture, Milling, Cad-cam, Complete denture

## Abstract

**Background:**

Historically, the complete removable denture is the last prosthetic procedure to switch to digital techniques whose advantages are mainly observed in the laboratory stages; however, it is not possible to measure the depressibility of the oral mucosa using optical cameras, thus conventional impression techniques are still necessary. This article describes the clinical and laboratory procedure and practitioners appraisal of the first fifteen digitally designed complete removable dental prostheses.

**Methods:**

Several systems are now available including the Wieland® Digital Denture® which offers a complete procedure. This system is composed of a five axis-milling machine combined with a laboratory scanner and a design software application. Fifteen rehabilitations were carried out using the Wieland® system.

**Results:**

The practitioner’s role is simplified by intraoral recording with a central point and a reduced number of sessions. The prosthesis laboratory requires considerable investment in learning and equipment, making it possible to obtain ideal mounting assemblies in accordance with the occluso-prosthetic concept of bilateral balanced occlusion. The absence of polymerization and therefore of base deformation risks reduce the equilibration step. Finally, the creation of templates as an alternative to the assembly of teeth on wax makes it possible to functionally validate (masticatory and phonatory) the future dentures. However, this procedure still presented some limitations in terms of scanning and software scope of applications**.**

**Conclusion:**

Digital denture design software is relatively efficient and helps to standardize clinical results. However, to this date, improvements of the software are still required for a routine use.

## Background

In recent years, technological advances have facilitated the development of Computer Aided Design and Manufacturing (CAD/CAM) and decreased the inaccuracies of conventional techniques [1]. Consequently, the evolution of digital systems and the development of chair-side systems have led to the integration of CAD/CAM in the therapeutic arsenal of fixed prosthesis procedures [2]. However, removable denture design has used the same “classical” procedures for more than fifty years despite their being associated with many risks of errors [3–5]. Thus the advantages of setting up a digital chain seemed obvious. Recently, procedures have been proposed for Removable Partial Dentures but they have remained on partially described and lacked substantial follow-up studies [6–8]. The complete removable denture procedure was the last method practiced before switching to digital technology. Historically, the first experimental works published date back to the mid-1990s [9]. Although several systems are now available and have been described in the literature [6–8], their usage by dental practitioners and/or technicians remains marginal on a daily basis. This work aims at describing the alternative options offered by CAD/CAM Complete Removable Denture systems compared to conventional methods. Within this context, fifteen rehabilitations were carried out using the Wieland® system. Their clinical evaluations were used to define whether transition to digital technology really contributed to enhancing and facilitating procedures for practitioners, prosthetists and complete denture wearers.

## Methods

In 2016, the acquisition of the Wieland® Digital Denture® system by the hospital dental unit enabled the realization of fifteen bi-maxillary complete dentures. This system can only run for complete edentulous patients. A major update of the system allowing uni-arcade denture processing is in progress. This system is composed of a five axis-milling machine combined with a laboratory scanner and design software (3shape™). Consequently, it offers a complete procedure which enables the practitioner to carry out all the clinical and laboratory steps. Thus the operators maintain an objective view and control over the digital procedure. This chain can be divided into four clinical sessions alternating with three dental laboratory stages. The iconographies presented come from different clinical cases. This report was approved by the local specific committee for the validation of professional practices analyses (A-93/03–2017).

### Clinical step 1

**Fig. 1:**
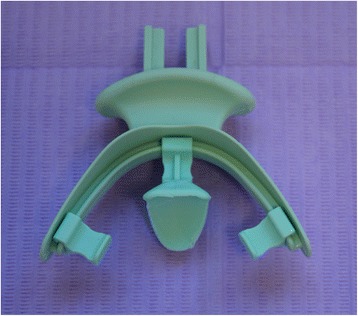
Centric Tray®

**Fig. 2:**
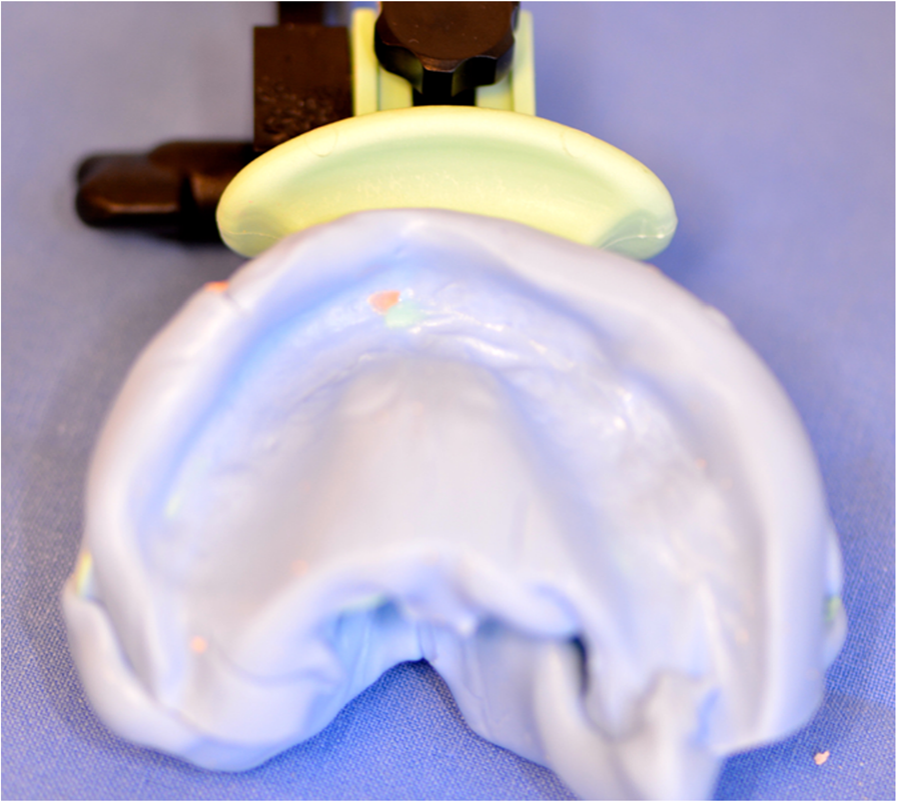
Recording of preliminary inter-arch report with the Centric Tray® and high viscosity elastomer

**Fig. 3:**
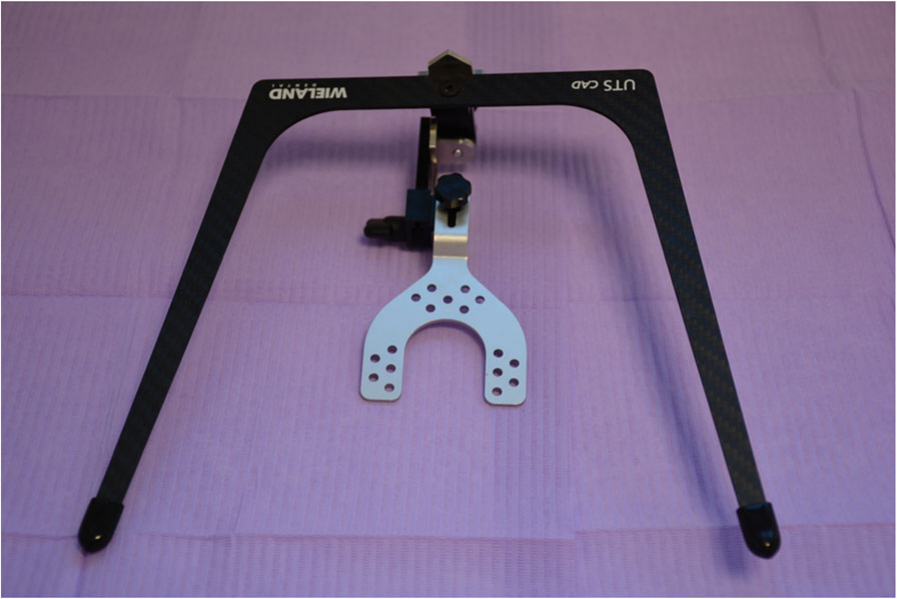
UTS CAD®

**Fig. 4:**
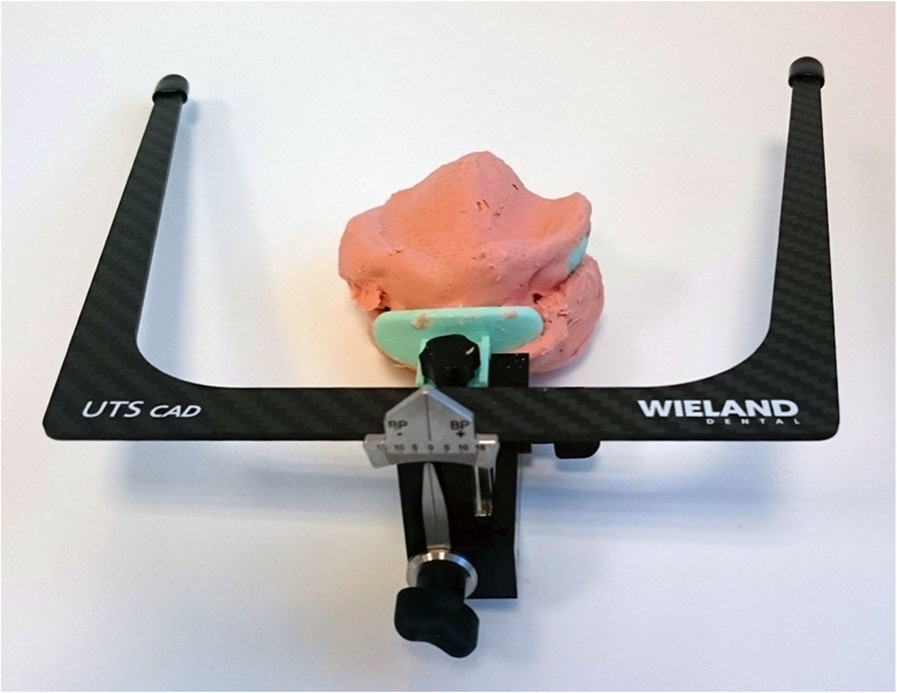
UTS CAD® device connected to the Centric Tray® for recording deviations from the reference planes

**Fig. 5:**
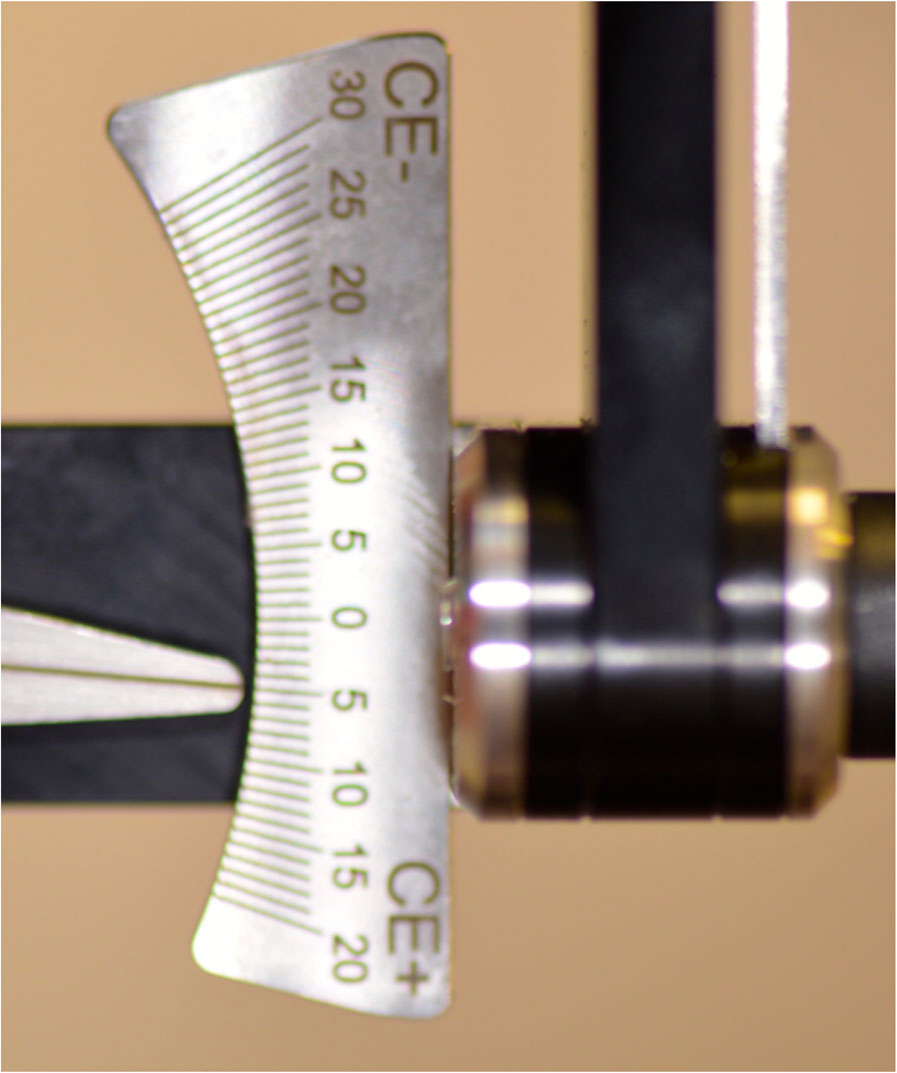
Measure of deviation from sagittal Camper plan

**Fig. 6:**
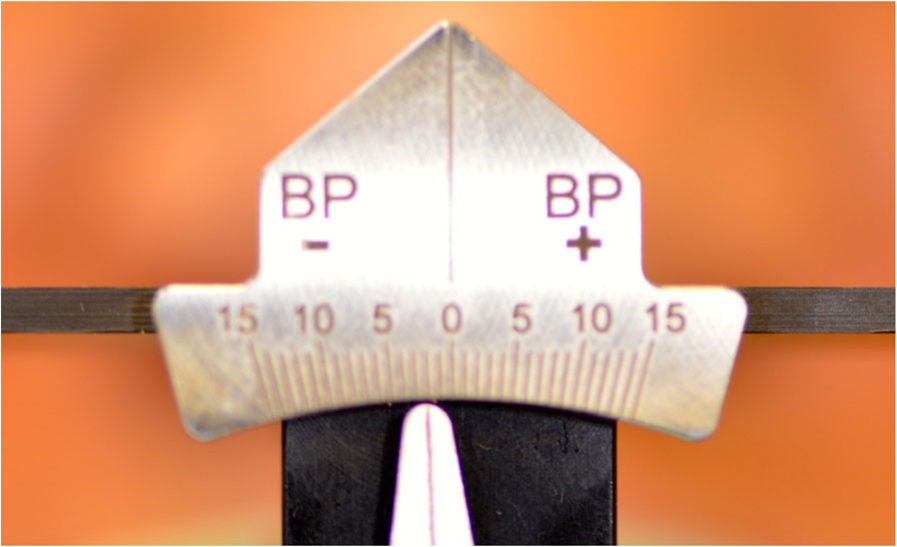
Measure of deviation from frontal bi-pupillary plan

The first clinical session of denture design consists of three stages: (1) Conventional physico-chemical (with plaster or alginate) maxillary and mandibular primary impressions. Optical impression of the edentulous arch can be performed but its implementation remains time-consuming and its advantages have remained limited up to now; (2) recording of the preliminary inter-arch report by a specific device (Centric Tray®, Ivoclar-Vivadent) (Fig. 1). This recording can be performed with alginate or high viscosity silicon (Fig. 2); and (3) the UTS CAD® device (Fig. 3) is used as a Fox plate to measure deviations from the reference planes (Sagittal Camper and bi-pupillary frontal planes). This device is fixed to the Centric Tray® (Fig. 4) and digital deviation values are measured (Figs. 5 and 6) and subsequently transferred to a virtual articulator. The main objective of this data recording step is to position the virtual primary models in space.

### Laboratory step 1

**Fig. 7:**
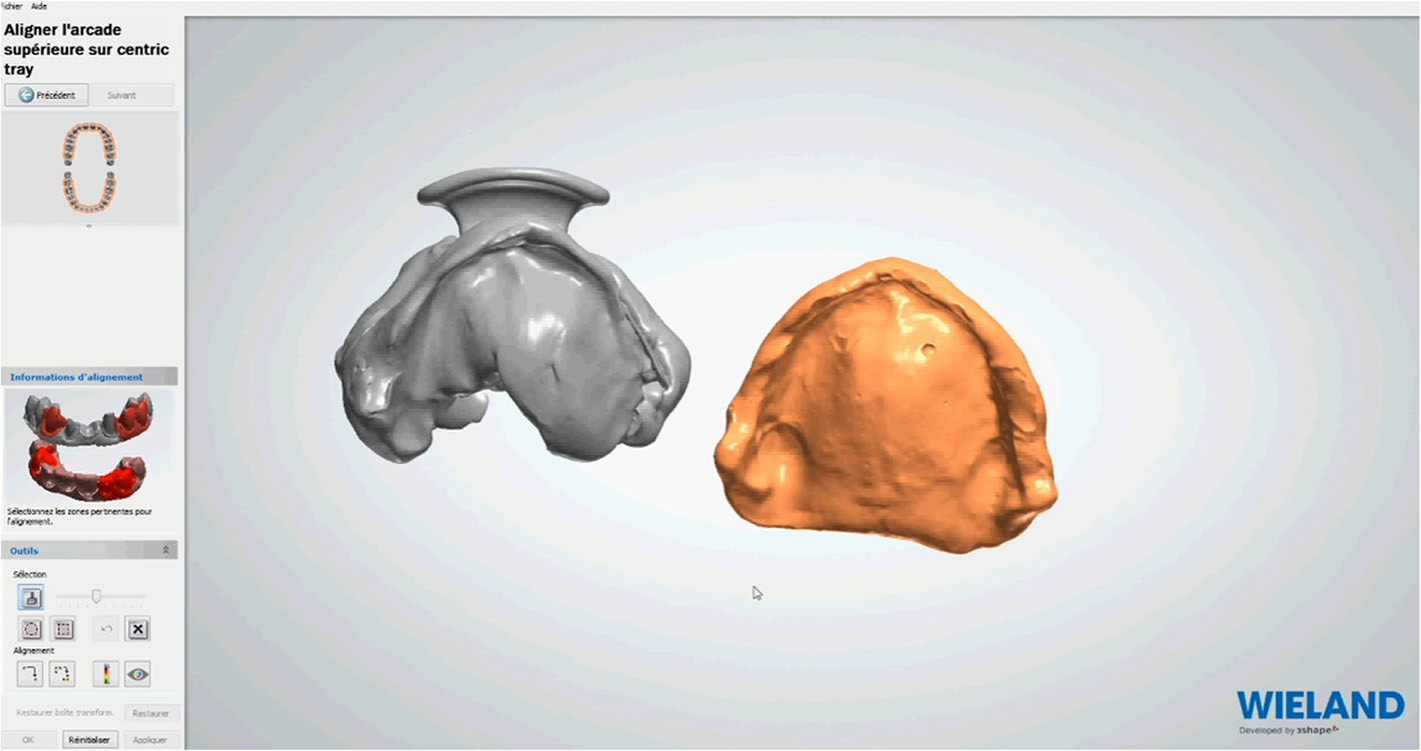
Superimposition of Centric Tray® bound impressions with primary impressions

**Fig. 8:**
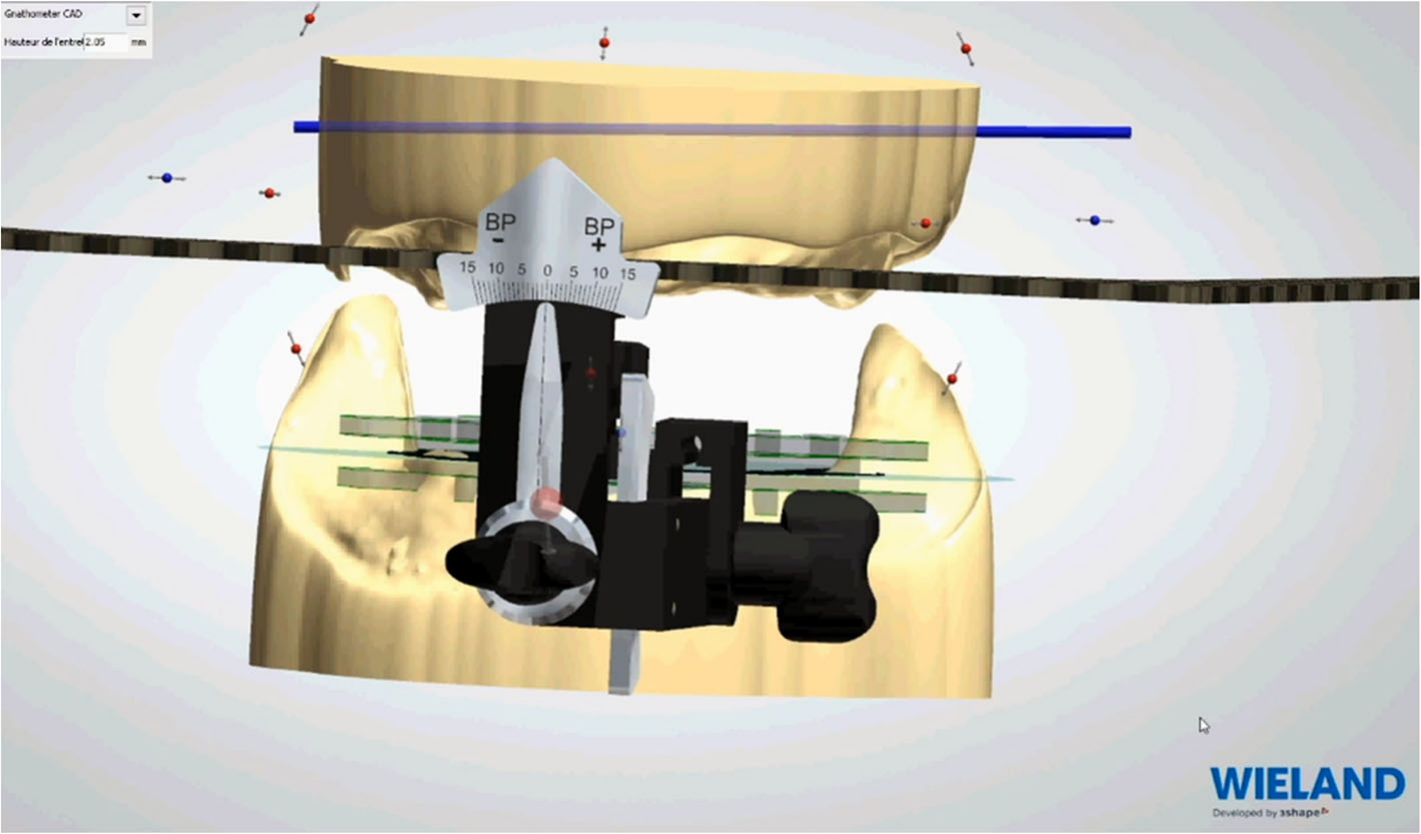
Values of the UTS CAD® device are used to create primary digital model positioned on a virtual articulator

**Fig. 9:**
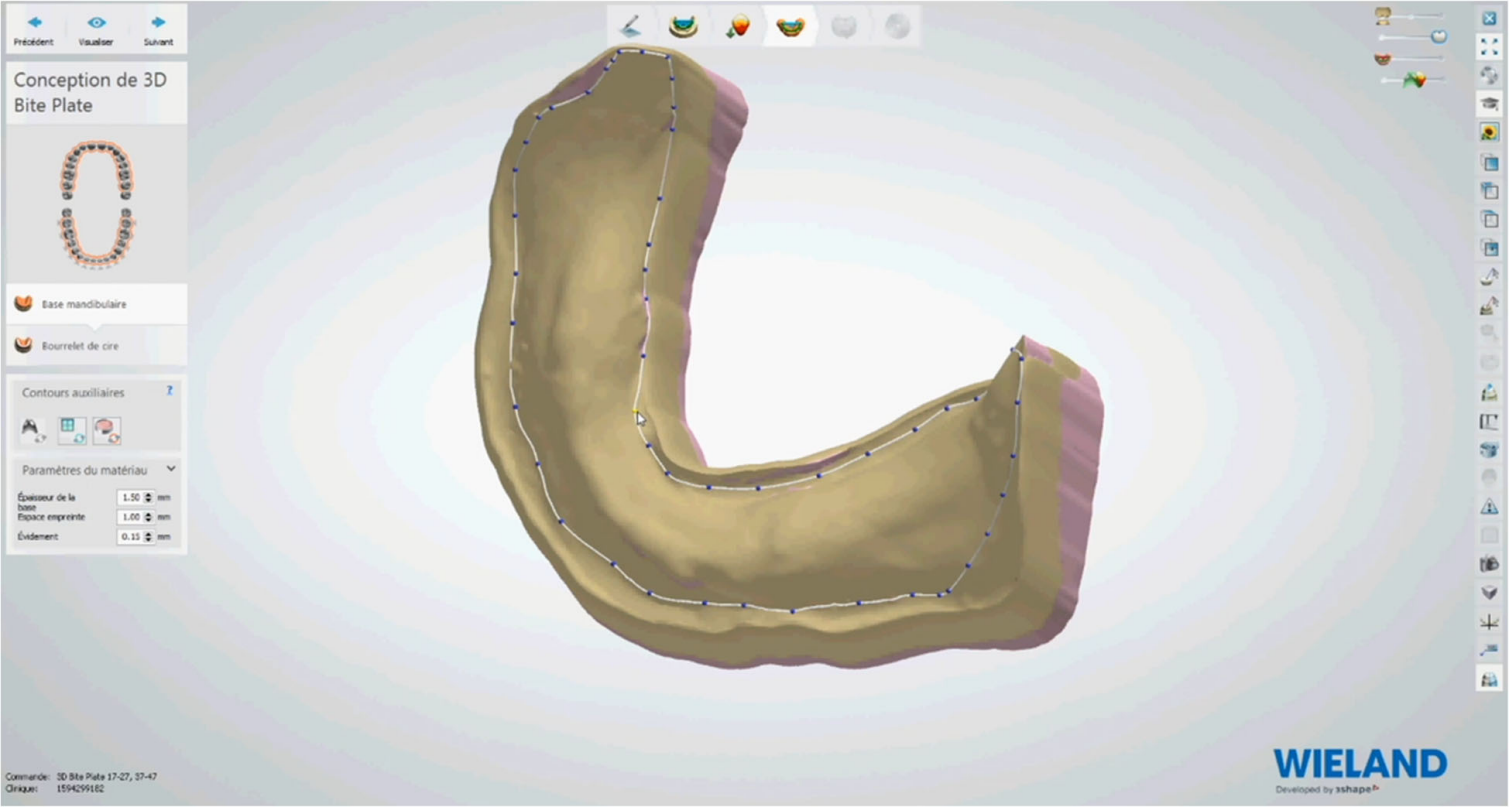
The edges of the individual trays are drawn according to conventional recommendations

**Fig. 10:**
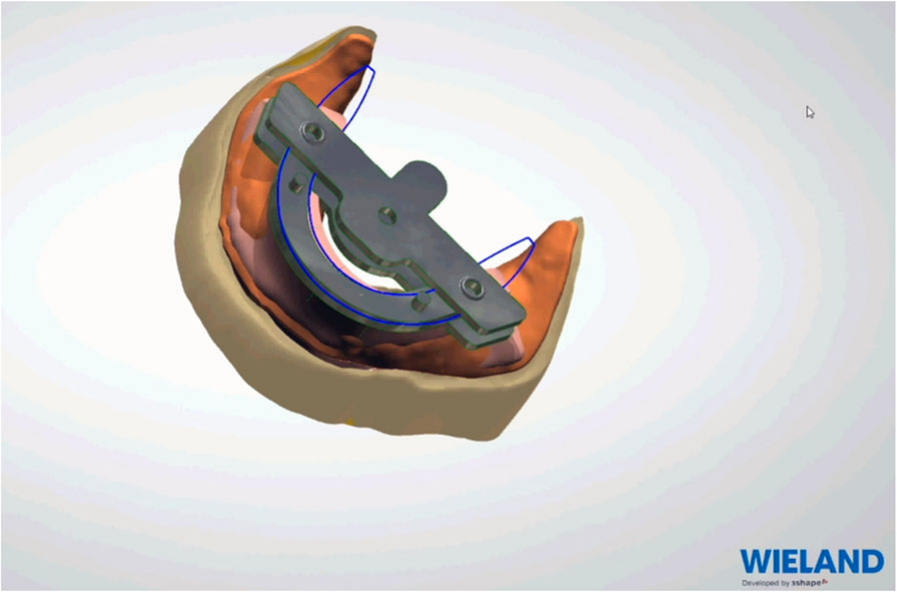
A cutback is integrated to leave sufficient space for the intra-oral center-point recording system (Gnathometer®) and to avoid any interference between the antagonist occlusal rims

Impressions bound around the Centric Tray® are scanned. Superimpositions of Centric Tray® bound impressions with the primary impressions (Fig. 7) and values of the UTS CAD® are used to create primary digital models positioned on the virtual articulator (Fig. 8). Thereafter, the edges of the individual trays are drawn according to conventional recommendations (Fig. 9). A specific software application (3shape) proposed a design for the occlusal rims, which are deliberately reduced in height compared to the dimension of the recorded vertical occlusion. A cutback is integrated to leave sufficient space for the intra-oral center-point recording system (Gnathomètre®) and to avoid any interference between the antagonist occlusal rims (Fig. 10). Once the design has been finalized, the project files are sent to the milling machine. The Wieland system comprises two 5-axis machine tools: the Zenotec Select Ion and the Zenotec Select Hybrid. The first is designed only for dry drilling of wax and PMMA (polymethylmethacrylate) resin. It includes air ionizers that facilitate cleaning due to the absence of electrostatic charges in the PMMA particles. The second is used for both dry and irrigated milling. Thus it can be used to drill glass-ceramics (Emax, Empress, etc.) and zirconia. Both machines have an 8-disc loader and do not require any external intervention during the milling step. A new range of milling machines might be proposed by Ivoclar-Vivadent in the near future.

### Clinical step 2

**Fig. 11:**
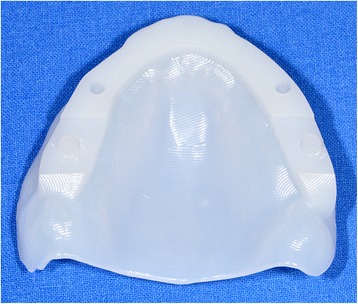
Maxillary individual milled tray

**Fig. 12:**
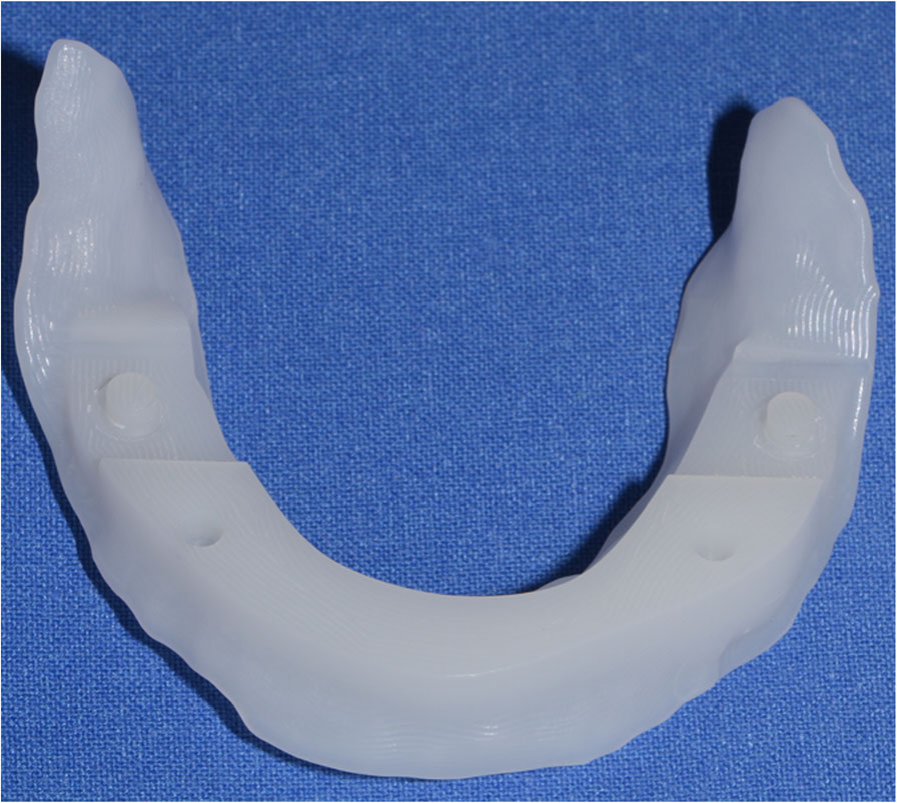
Mandibular individual milled tray

**Fig. 13:**
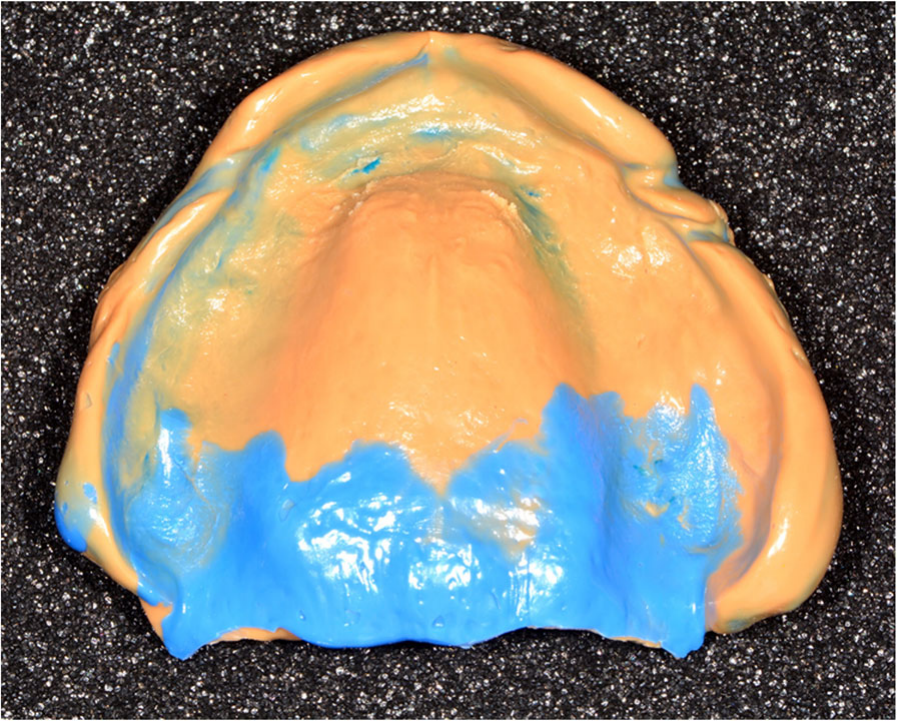
Maxillary conventional functional impression with the manufactured tray

**Fig. 14:**
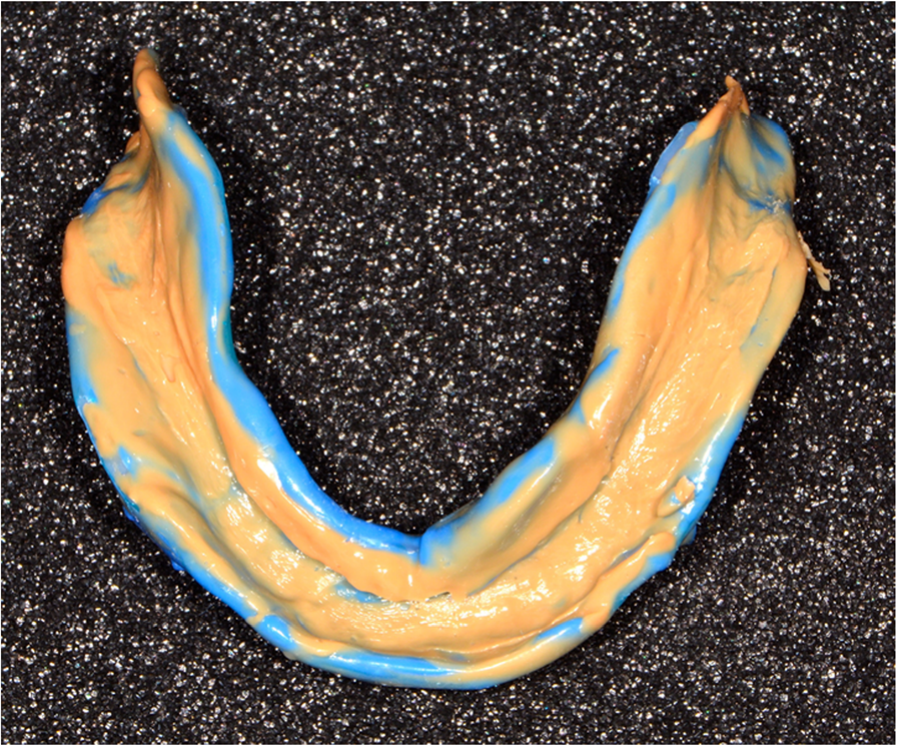
Mandibular conventional functional impression with the manufactured tray

**Fig. 15:**
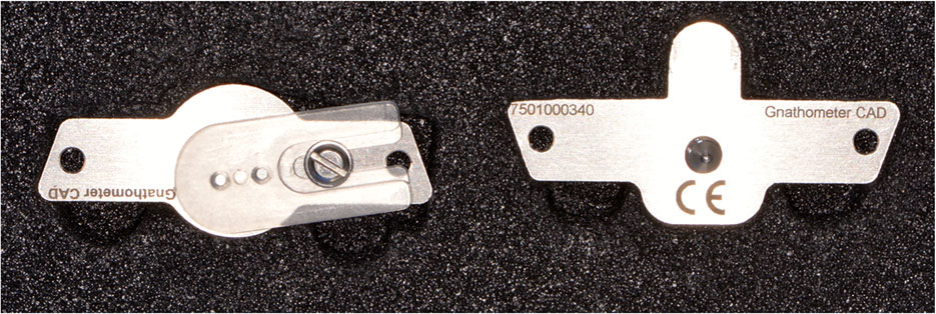
Gnathometer®

**Fig. 16:**
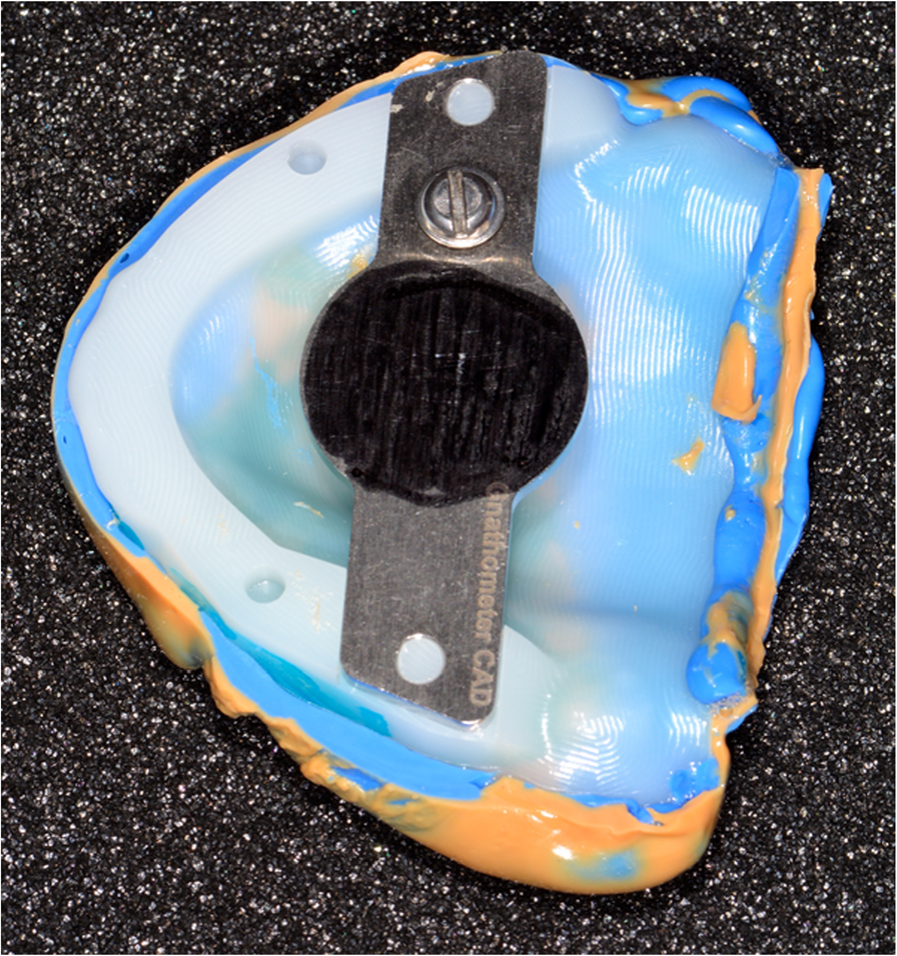
Gnathometer® can be easily clipped to tray impressions and does not require bonding or retention

Conventional functional impressions are realized with the manufacturing trays (Figs. 11 and 12), as the depressible nature of the mucosa cannot yet be recorded by optical impression (Figs. 13 and 14). These impressions will be introduced several times in the mouth. Consequently, the impression material contains some elastic component. The inter-arch relationship could be recorded in the same session. Before clipping the center-point system to the impression trays, the maxillary occlusal rim can be checked with UTS CAD® and its provided fork (Fig. 3). Also, an intraoral system with a central supporting point is used (Gnathometer® (Fig. 15)). A screwing/unscrewing pointer is used to modulate the occlusal vertical dimension. A circular receiving plate with a marker material enables registering the different mandibular movements. Gnathometer® can be easily clipped to tray impressions and does not require bonding or retention (Fig. 16).

**Fig. 17:**
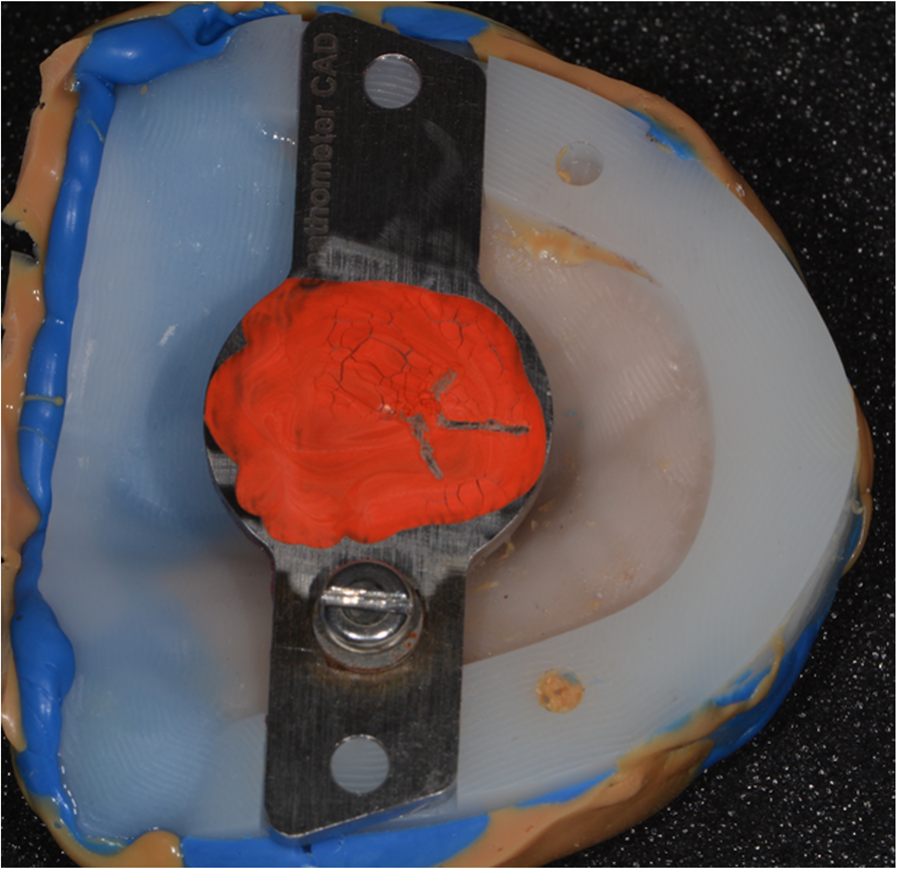
Gysi Gothic arch registered with Gnathometer®

**Fig. 18:**
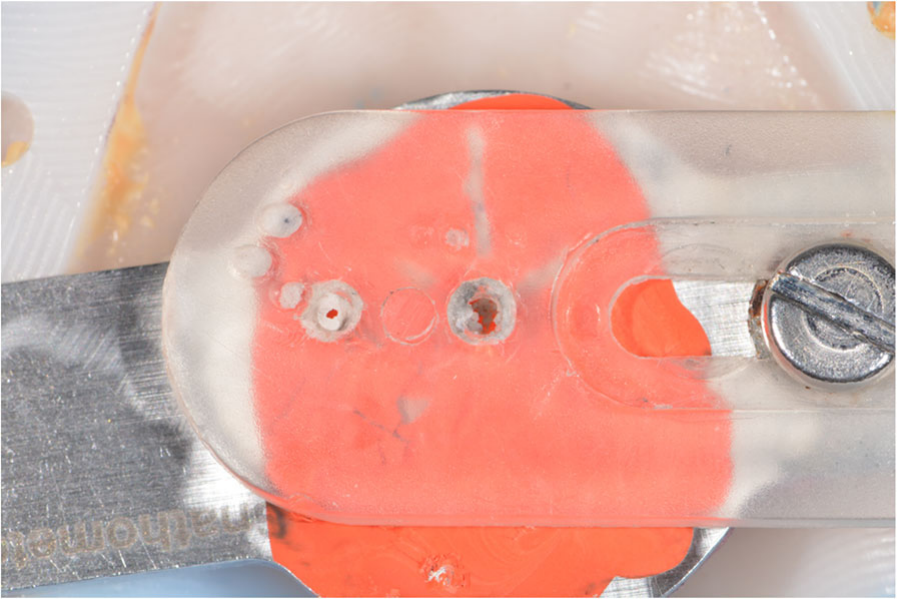
Gysi Gothic arch paths meet at an equilibrium area used as a reference during the inter-arch relationship recording step

**Fig. 19:**
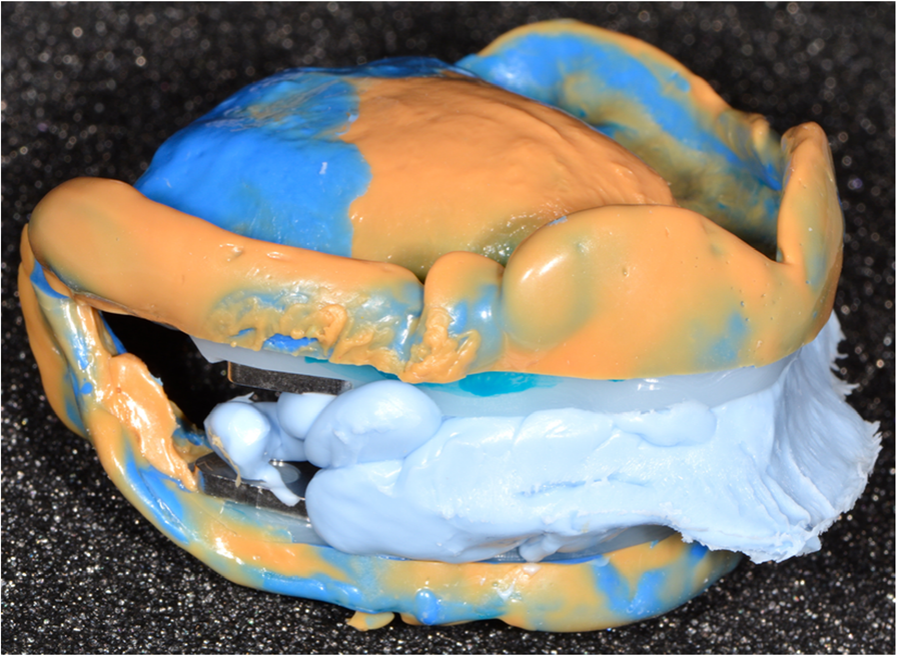
The use of elastomer material (silicone bites) allows shaping the lip support and locating the horizontal joint line of the lips

Next, the Occlusal Vertical Dimension (OVD) is determined and adjusted by screwing or unscrewing the central pointer. Modifications of the OVD are easy to achieve as the only contact between the maxilla and the mandible is made through the central pointer. This process avoids any interference from the occlusal rims, a likely source of mandible deviation, and allows excellent retention of the impression trays on the ridges. Then, the cylindrical receiving plate of the central pointer is tinted to obtain the mandibular paths (Gysi Gothic arch) (Fig. 17). These paths meet at an equilibrium area used as a reference during the inter-arch relationship recording step (Fig. 18). The use of elastomer material (silicone bites) allows shaping the lip support and locating the horizontal joint line of the lips (Fig. 19).

### Laboratory step 2

**Fig. 20:**
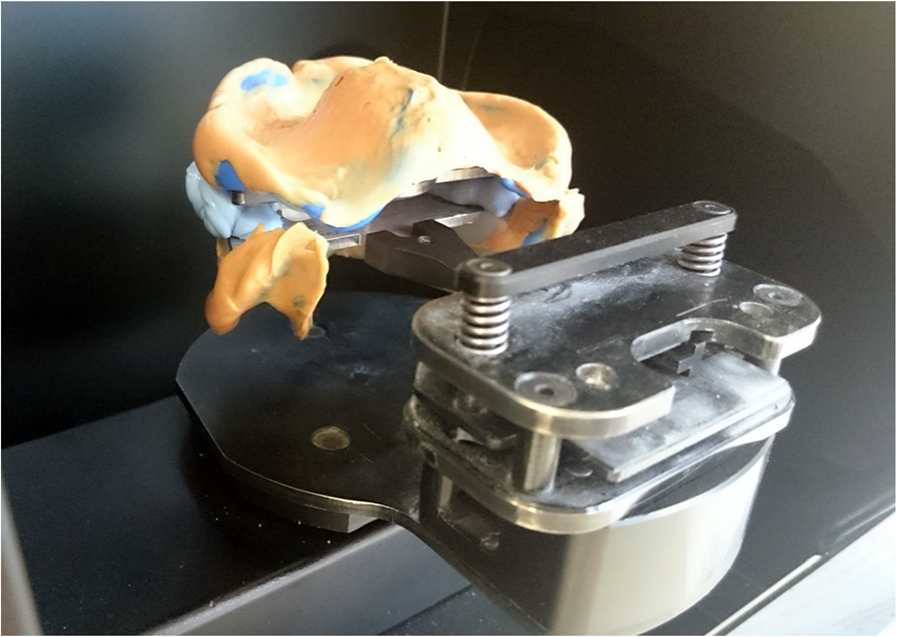
The embedded functional impressions are scanned

**Fig. 21:**
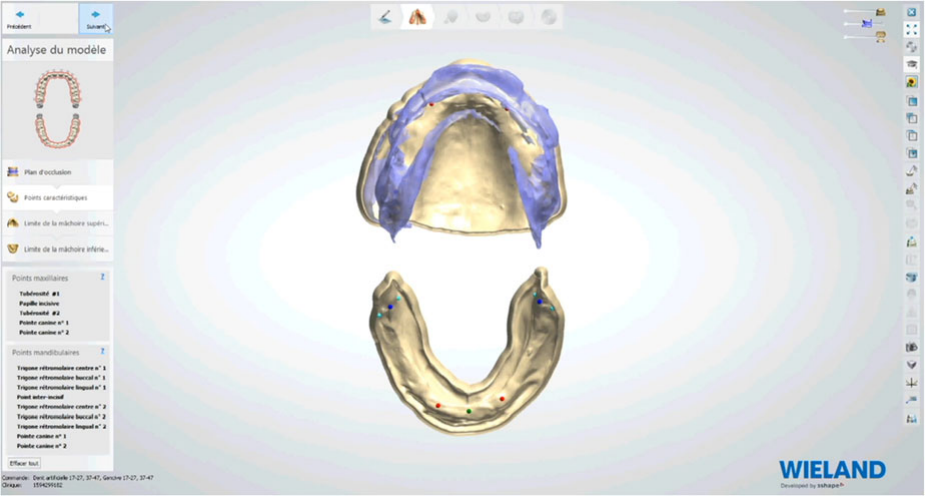
Numerical models are placed on the virtual articulator and identified reference points

**Fig. 22:**
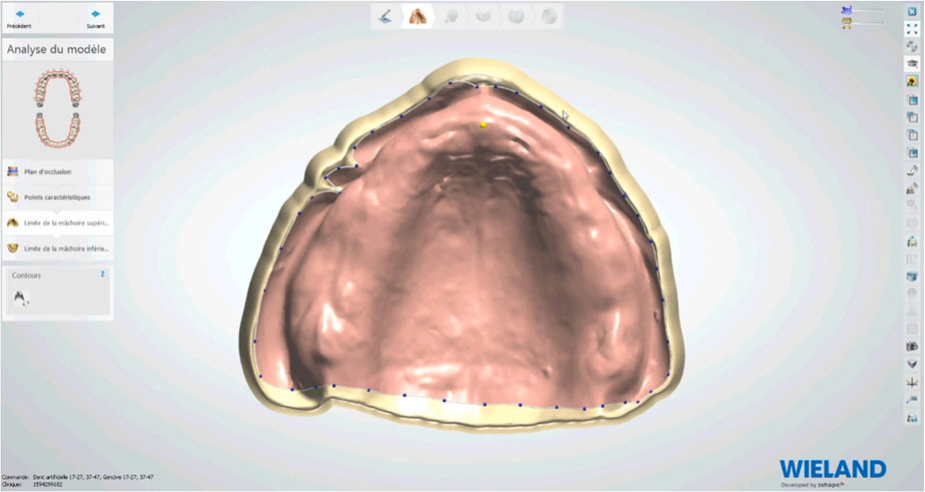
Drawing of the limit of the future denture base

**Fig. 23:**
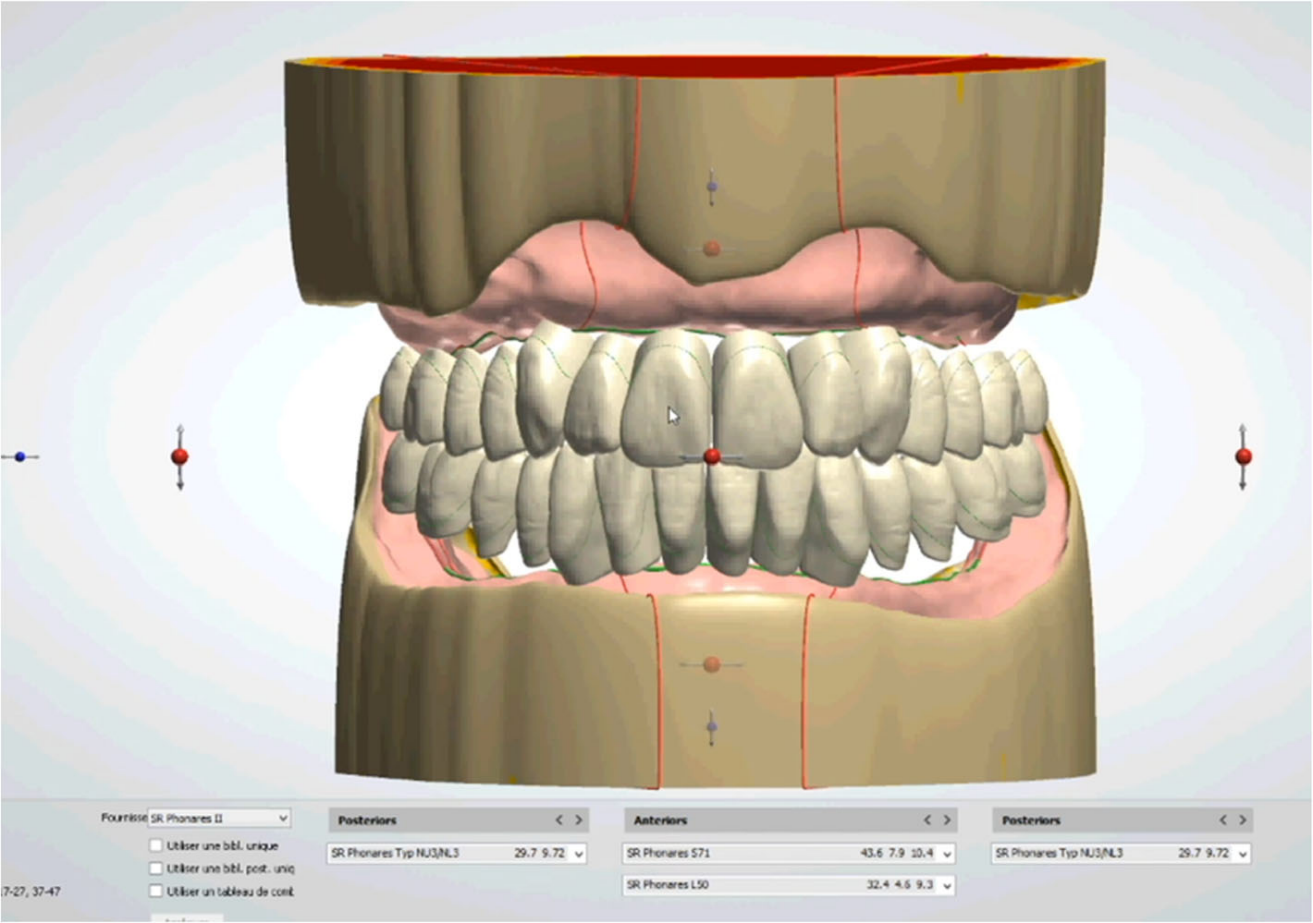
A teeth setting is proposed by the 3shape software with posterior teeth positioned in an ideally bilateral balanced occlusion concept

**Fig. 24:**
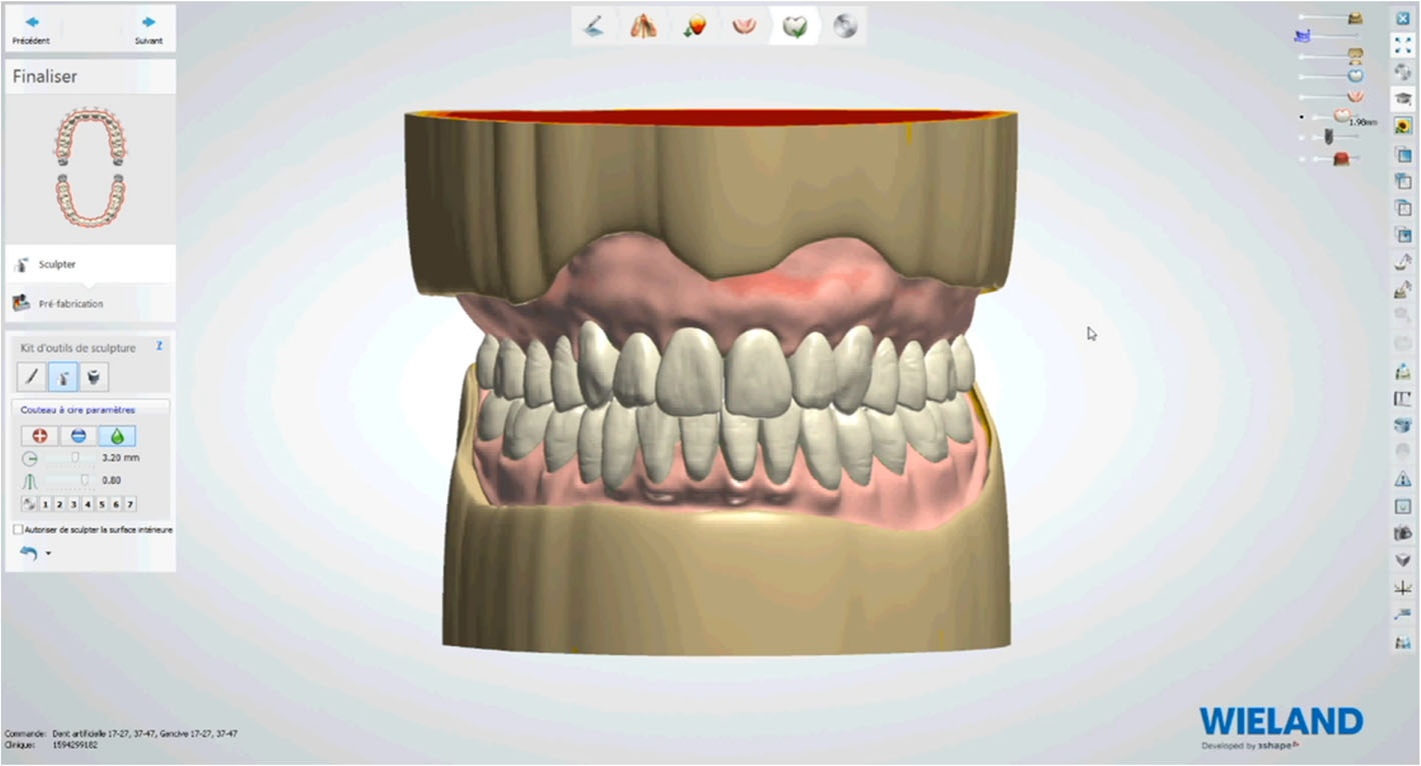
Virtual waxes finishing step

Afterwards, the embedded functional impressions are scanned (Fig. 20) to obtain the virtual working models. These models are placed on the virtual articulator and the reference points are identified (incisal papilla, canine tooth tips, retro-molar pad centers, limit of the retro-molar pads, tuberosities, etc.) to trace a schematic representation of the Pound area for posterior teeth positioning (Fig. 21). Then, the limit of the future denture base is drawn (Fig. 22) and a teeth setting is proposed by the 3shape software (Fig. 23). The software contains a library of teeth of different brands and shapes and a function with an automatic proportional size table between the anterior and posterior teeth. Subsequently, the operator is able to modify every parameter except those for tooth removal. At present, only second molars can be removed, in the case of short arches. The solution proposed by the software for the positioning of the posterior teeth is ideally suited for the morphology of prosthetic teeth, hence facilitating the integration of a bilateral balanced occlusion concept. Once this assembly has been validated, finishing of the virtual waxes must be carried out (Fig. 24) to avoid imperfections that could later prevent milling: the software does not indicate these future problems during the design phase.

**Fig. 25:**
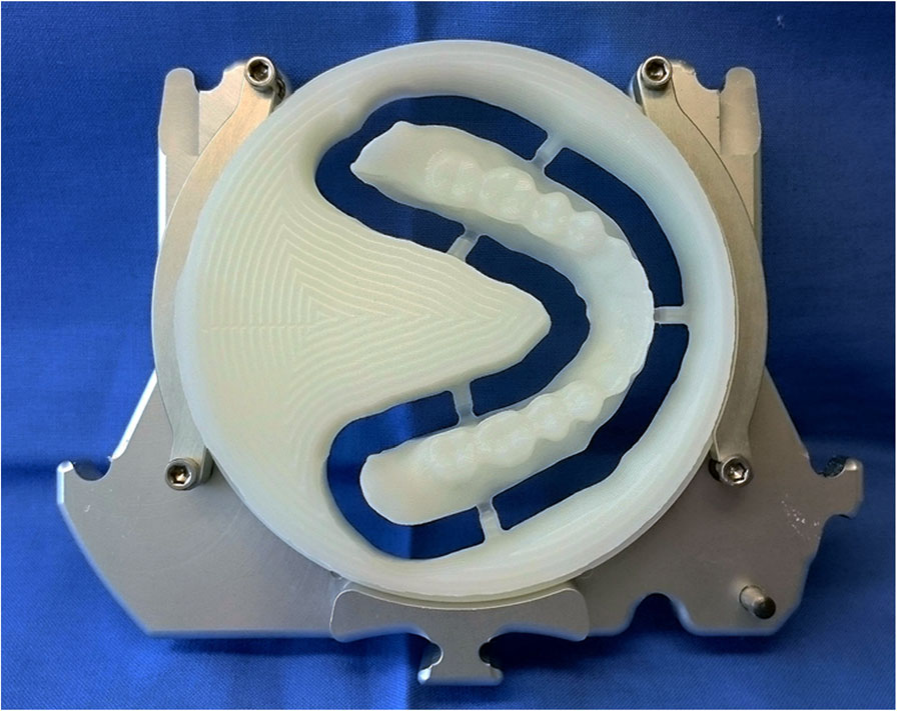
Manufactured template on a white PMMA disc

**Fig. 26:**
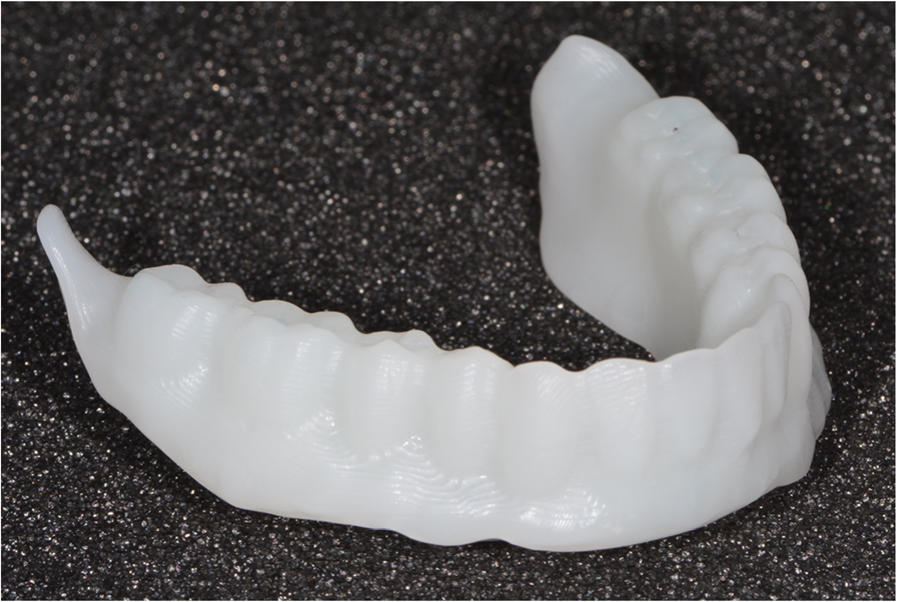
Mandibular template

**Fig. 27:**
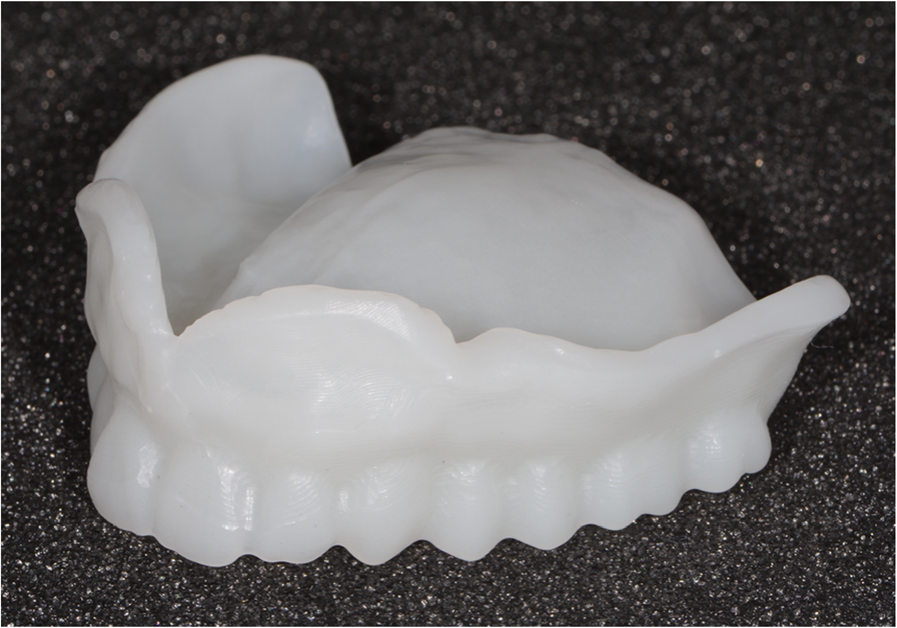
Maxillary template

After reception of the files by the command software of the milling machine (Wieland), the complete denture project (denture bases and prosthetic teeth) is manufactured on a white PMMA disc (Figs. 25, 26 and 27), leading to the production of a much more useful template than when obtained with the conventional method.

### Clinical step 3

**Fig. 28:**
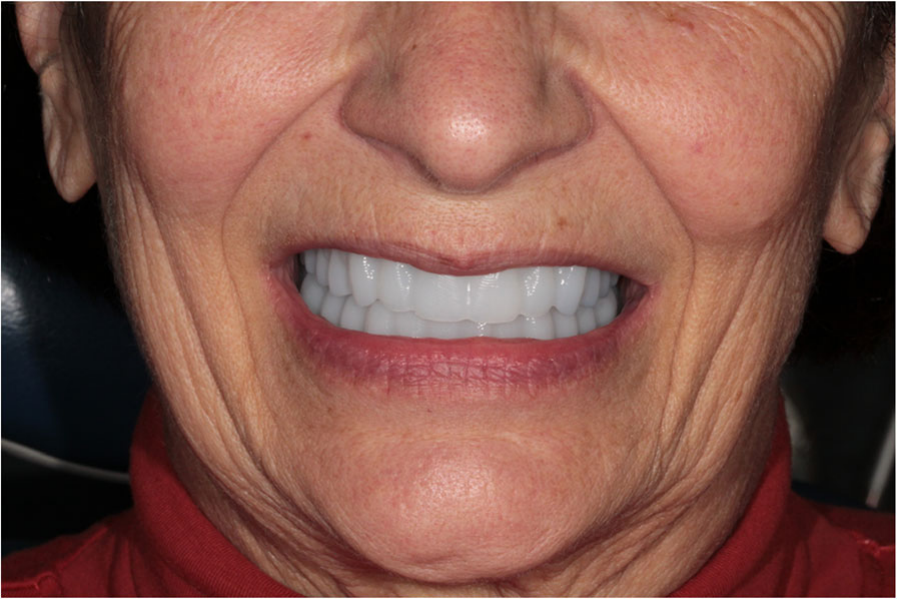
The occlusion rims are validated and patients use their manufactured template for functional validation (mastication and phonation) for a while at home

**Fig. 29:**
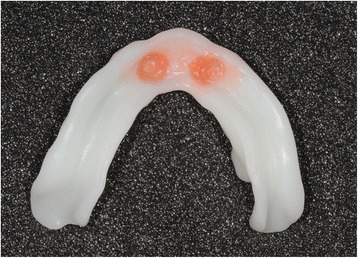
A retention silicone (Retention.Sil® Bredent®) can be used to replace the intra-prosthetic retention part if the patient already wears complementary retention system

During this session, the occlusion rims are validated and patients use their manufactured template for functional validation (masticatory and phonatory) for a while at home (Fig. 28). If the patient already wears complementary retention systems (roots or overdenture implants), a retention silicone (for example: Retention.Sil® Bredent®) can be used to replace the intra-prosthetic retention part (Fig. 29). Similarly, if the patient has neuro-musculo-articular disorders detected during the clinical examination or the recording of the Gysi Gothic Arc, this template can provide a cheaper rehabilitation solution. In addition, these templates can easily be used as radiological and / or implant guides for a subsequent prosthetic project. After the try-out period, the patient’s criticisms are collected and modifications are made if necessary. In cases of significant changes, new templates can be machined. The final step can be initialised after validation of the functional aspect.

### Laboratory step 3

**Fig. 30:**
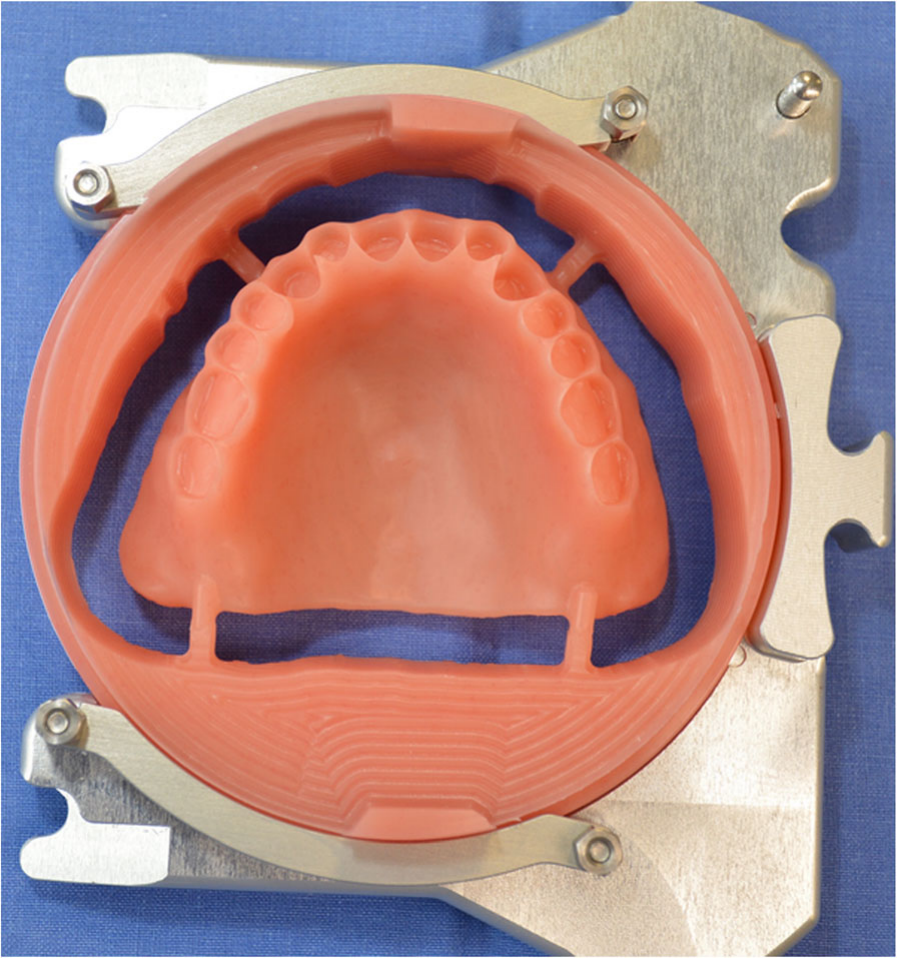
Milling of the denture extrados on a pink resin disc with specific alveoli for the prosthetic teeth, depending on the brand and model of the teeth selected

**Fig. 31:**
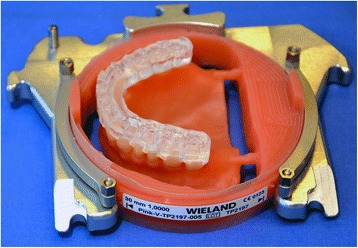
A positioning key is milled to ensure the ideal setting of the teeth during the bonding process with a PMMA resin

**Fig. 32:**
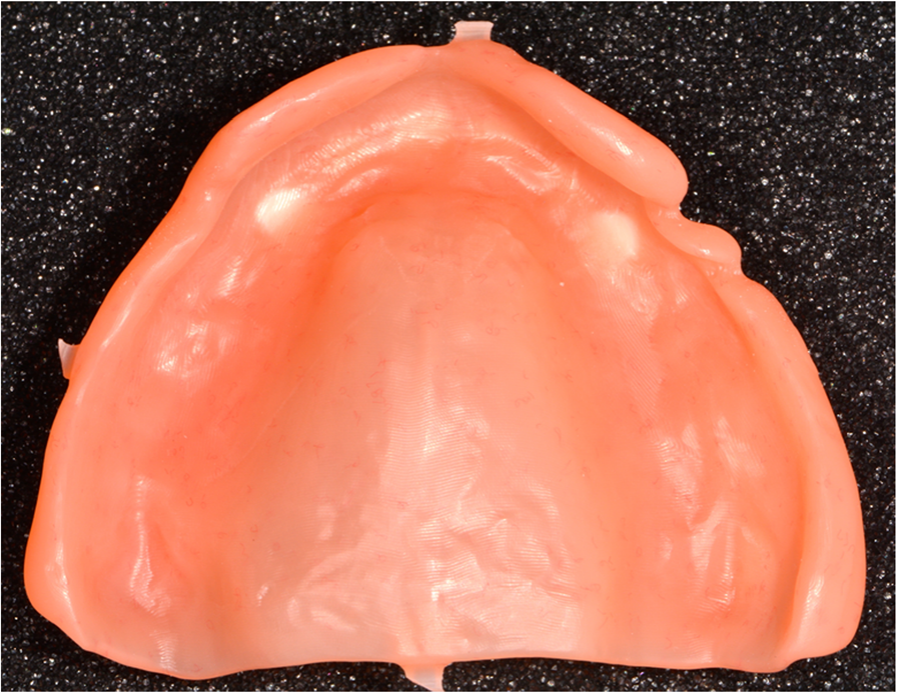
Once the bonding is complete, the disc is put back into the machine to mill the denture intrados. If a prosthetic tooth base interferes with the virtual model, it will be machined according to the correct intrados

Four steps are necessary to complete the denture: (1 and 2) milling of the denture extrados on a pink resin disc with specific alveoli for the prosthetic teeth, depending on the brand and model of the teeth selected (Fig. 30). Thereafter, a positioning key is milled to ensure the ideal setting of the teeth during the bonding process with a PMMA resin (Fig. 31). (3) Once the bonding is complete, the disc is put back into the machine to mill the denture intrados. If a prosthetic tooth base interferes with the virtual model, it will be machined according to the correct intrados (Fig. 32) (4). The denture is removed from the disc, scraped and polished according to the conventional procedure. It should be noted that the surface finish after machining is very satisfactory.

### Clinical step 4

**Fig. 33:**
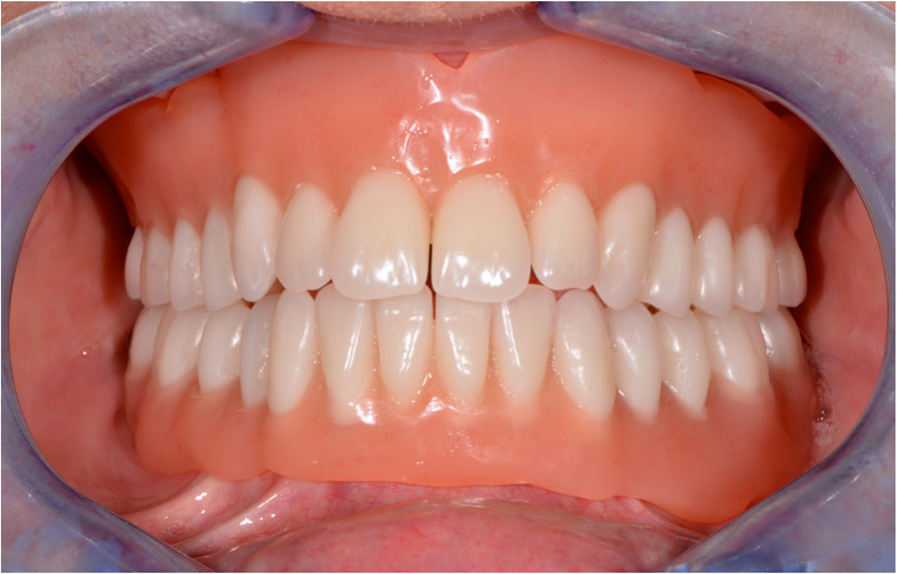
The dentures are tried and primary equilibration is performed

**Fig. 34:**
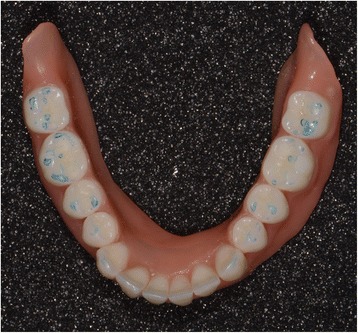
First equilibration session, as all steps are validated

During this last session, the dentures are tried and primary equilibration is performed (Fig. 33). If all the steps have been conformed to and validated, the first equilibration session is often unnecessary or very easy (Fig. 34).

## Results

Follow-up assessment of the complete denture rehabilitation was performed for the first fifteen subjects (65 ± 13 years, 48 to 81 years old, 7 women and 8 men). They included eight Class II subjects. The subjects’ complete dentures underwent minor corrections during the follow up sessions. For two subjects, the vertical dimension was observed as overestimated during the template wear try-out period and corrected. This series of clinical cases could be used to establish a preliminary comparison with conventional complete dentures (Table 1) and pave the way to interesting future prospects.

**Table 1:** Assessment of digital complete denture versus conventional denture based on the first fifteen rehabilitations

Advantage of CAD/CAM procedure	Digital denture vs conventional procedures	Results
Facilitating system?	Number of clinical sessions	Decrease
	Repeatability of recordings	Better
	Systematic procedure reliability	Verified
	Suitability for All Angle Class	Designed only for CL I
	Material durability	Maybe, follow-up studies needed
Occlusal Accuracy?	Occlusion Transfer	Better
Profitable system?	Cost /efficiency ratio	Maybe, socio-economic studies needed
Physiological interest?	Functional Training	Yes, temporary usable template (Prior final validation)
Data digitization?	Data backup/storage	Yes

However, this procedure still presented some limitations. Indeed, the scan and modeling phase still brought about great difficulties. On one hand, the scan surface was not sufficient enough to totally register the impression components: it was not possible to scan trigon and para-lingual areas simultaneously, or tuberosity and para-tuberosity areas. On the other hand, the fixation method (with fixating paste) of the impression tray on the magnetized scanner plates remained artisanal. These recurring episodes induced an important working time delay (4 or 5 time more) compared to a classic straightforward scan and design phase, and remained longer compared with the time required for traditional impressions treatment.

In addition, the designing phase was far from matching the manual work of a skilled laboratory prosthesis technician, as it was not possible, for example, to choose to remove one or several teeth depending on the wanted result, to finish waxing of the polished functional edges, to have adaptable thickness, minimal and distinct on different parts of the tray, to specify the position of the teeth mounting plan, or to choose the type of mounting with the different exploitable correction curves. New updates should correct these problems.

### Discussion

This first study was performed to evaluate and to validate the clinical and laboratory processes and their potential integration into a daily practice. The evaluation of the treatment success was not a primary goal; to this end, follow-up controls must be planned for each patient over time.

The Complete Removable Denture is the last prosthetic procedure to switch to digital techniques whose advantages are mainly observed in the laboratory stages; however, it is not possible to measure the depressibility of the oral mucosa using optical cameras, thus conventional impression techniques are still necessary. However, the complete innovative Wieland® system allows standardizing procedures and fewer sessions for the practitioner. A central point intra-oral recording system can be used to ensure complete reproducibility of the inter-arch ratio. This new system is beneficial for producing complete dentures, a domain of prosthetics that is often neglected and sometimes frustrating, as the results obtained do not always live up to the expectations of the practitioner or dental technician.

A recent study showed that the milling process is more advantageous in terms of retention compared to the conventional polymerization procedure [10]. The absence of polymerization prevents distortions of the bases and teeth displacement. The teeth positioning key allows precise occlusion placement, programmed by the software. The virtual mounting of the posterior teeth is therefore ideally suited to the morphology of the prosthetic teeth, facilitating the integration of a bilateral balanced occlusion concept. This constitutes a major improvement in comparison to manual positioning. Also, this logistic evolution facilitates the dental laboratory step in which occlusal concepts and denture mountings require highly specific technical skills. Until now, these competences in the domain of complete dentures were considered as less important than implant or esthetic treatments.

As in other disciplines, the CAD/CAM procedure for complete dentures should increase the level of realizations. However, the investment in both learning and equipment remains considerable for dental laboratories. In this sense, investing in the Zenotec Select Hybrid is an alternative solution for extending prosthetic work (fixed and prosthetic implants). Virtual modeling requires training as 3D visualization on screen could be disconcerting and cause difficulties when reading the final result, especially during the wax finishing step. Cutting the mandibular impression, in particular in the retromolar pad area, is delicate as its contact surface must be optimal for ideal fitting. Indeed, removing a tooth next to the second molar is impossible, making it difficult to mount dentures in the case of Class II subjects. According to the supplier, this digital procedure is only intended to treat Angle Class I subjects. However, in the dental hospital unit, numerous Class II subjects were treated: the possibility of removing a premolar represents a crucial improvement of the software. As of today, the only available option to treat patient with an Ackermann class different from class I is either the removal of the second molar or changing the size of the prosthetic teeth without using matching tables between the anterior teeth and the posterior teeth. One improvement of the software would be to allow removal of one or multiple teeth to facilitate treatment of class II and III patients or to set up an overjet.

The correct positioning of the plate for recording the Gothic Arch is indispensable, which is facilitated by the snap-in system procedure. The height of the occlusal rim, determined by the measurements recorded with the Centry Tray, is undervalued to avoid interference between the two arches. The orientation of the occlusal rim is adjusted using the values recorded with the UTS CAD. One of the limiting factors is the arcade shape which does not allow the snap-fit system to be placed on the rim because only one Gnathometer dimension is available today. Severe class II or too narrow arcades will require a traditional recording system of the inter-arcade relationship.

From a clinical point of view, if all the favorable conditions of realization are met, patients will require professional guidance for the registration of their mandibular movements.

Nonetheless, the capacity of the CAD/CAM procedure to deal with any individual tooth, with mounting assistance and simultaneous machining of several dentures without polymerization is an improvement leading to fewer errors encountered during the realization of Removable Dentures [3–5].

From the practitioner’s point of view, the digital denture procedure is timesaving since the clinical step of functional impression and recording of the inter-arch ratio are coupled. However there are difficulties in recording occlusion with the intra-oral central point with some patients. In addition, the practitioner must ensure that trays and retro-molar pads or tuberosity do not interfere with the recording. If difficulties occur, conventional recording can be carried out instead.

## Conclusion

Digital denture design software is relatively efficient and helps to standardize clinical results. However, the Wieland system is far from meeting the requirements necessary for a routine use, and much improvement are required, for example scanning surface should be increased. Furthermore, the software should be improved to be able to deal with more clinical cases. Indeed, the system is optimized for Class 1 subjects, while Class II Angle subjects are increasingly encountered in dental units. Consequently, finalization of the mounting is more difficult, all the more so because it is not possible to choose which tooth to remove to obtain the ideal occlusion. In the future, regular and physiological clinical assessment during follow-up sessions of a large number of cases would determine the interest of this new procedure, providing promising potential in the field of complete and removable dentures.

## Abbreviations

CAD/CAM: Computer Aided Design and Manufacturing
OVD: Occlusal Vertical Dimension
PMMA: Polymethylmethacrylate

## References

[1] Bohner LOL, Neto PT, Ahmed AS, Mori M, Laganá DC, Sesma NCEREC (2016). Chairside system to register and design the occlusion in restorative dentistry: a systematic literature review. J Esthet Restor Dent.

[2] Ahlholm P, Sipilä K, Vallittu P, Jakonen M, Kotiranta U. Digital versus conventional impressions in fixed prosthodontics: a review. J Prosthodont 2016; doi: 10.1111/jopr.12527. [Epub ahead of print]. 10.1111/jopr.12527 27483210

[3] Rudd RW, Rudd KDA (2001). Review of 243 errors possible during the fabrication of a removable partial denture: part I. J Prosthet Dent.

[4] Rudd RW, Rudd KDA (2001). Review of 243 errors possible during the fabrication of a removable partial denture: part II. J Prosthet Dent.

[5] Rudd RW, Rudd KDA (2001). Review of 243 errors possible during the fabrication of a removable partial denture: part III. J Prosthet Dent.

[6] Kattadiyil MT, Goodacre CJ, Baba NZ (2013). CAD/CAM complete dentures: a review of two commercial fabrication systems. J Calif Dent Assoc.

[7] Schwindling FS, Stober TA (2016). Comparison of two digital techniques for the fabrication of complete removable dental prostheses: a pilot clinical study. J Prosthet Dent.

[8] Kattadiyil MT, AlHelal A (2016). An update on computer-engineered complete dentures: a systematic review on clinical outcomes. J Prosthet Dent.

[9] Maeda Y, Minoura M, Tsutsumi S, Okada M, Nokubi T (1994). A CAD/CAM system for removable denture. Part I: fabrication of complete dentures. Int J Prosthodont.

[10] AlHelal A, AlRumaih HS, Kattadiyil MT, Baba NZ, Goodacre CJ (2016). Comparison of retention between maxillary milled and conventional denture bases: a clinical study. J Prosthet Dent.

